# Exploring Portuguese physicians' perceptions of climate change impacts on health: A qualitative study

**DOI:** 10.1016/j.joclim.2024.100333

**Published:** 2024-07-08

**Authors:** Nidia Ponte, Fátima Alves, Diogo Guedes Vidal

**Affiliations:** a Department of Social Sciences and Management, Universidade Aberta, 1250-100, Lisbon, Portugal; b Centre for Functional Ecology—Science for People and the Planet (CFE), Associate Laboratory TERRA, Department of Life Sciences (DCV), University of Coimbra (UC), 3000-456, Coimbra, Portugal; c Observatory of Sustainable and Healthy Territories of Bocaina, Paraty, 23970-000, RJ, Brazil; d Sergio Arouca National School of Public Health (Ensp), Oswaldo Cruz Foundation (Fiocruz), Rio de Janeiro, 21041-210, RJ, Brazil

**Keywords:** Physician's, Climate change, Health, Perception, portugal, qualitative, education, continuing medical education, environment

## Abstract

**Introduction:**

Health professionals, as primary responders to climate change health impacts, must be well-informed to effectively communicate adaptation risks and benefits to influence both patients and organizations. This study was undertaken in order to survey how physicians in Portugal understand, explain, and experience the impacts of climate change on health in their clinical practice. Understanding physicians' knowledge level on this subject also can help determine whether training is needed, and through what educational mechanisms.

**Material and methods:**

Using a semi-structured interview script, this study applied a qualitative methodology with interviews of 13 physicians from various medical specialties in Portugal, aged 28 to 73.

**Results:**

All participants recognized that human action is the main contributor to climate change, indicating that fossil fuels and overproduction are the main factors responsible for greenhouse gas emissions. They also agreed that climate change affects human health. However, they reported that they find connecting diseases to climate change challenging in their clinical practice, and expressed the need for training on the effects of climate change on health.

**Conclusion:**

This study demonstrated that physicians recognized the importance of understanding and communicating the connection between climate change and health in their daily practice, and that failure to recognize these impacts may affect appropriate diagnosis and preparedness for extreme climate events. These results highlight the urgency of addressing the health impacts of climate change and underscore the role of education and awareness.

## Highlights

• First study on Portuguese physicians' perceptions about climate change health impacts.
• Physicians recognize climate effects, notably increased respiratory issues.
• Limited knowledge exists on the climate-health link.
• There is a need to integrate climate awareness into medical training.

## 1 Introduction

Climate change's health impacts are escalating and intensifying with rising global temperatures, necessitating urgent research to emphasize these issues scientifically, politically, and technically. The Intergovernmental Panel on Climate Change (IPCC) forecasts a 2 °C temperature increase by 2050 without immediate mitigation, and urges governments to alter or enhance their climate change (CC) strategies, a challenge observed at various Conferences of the Parties (COPs) [1]. The gravity of the matter amplifies as CC affects health and presents equity challenges, disproportionately impacting children, the elderly, the chronically ill, and socio-economically vulnerable populations [2]. With its Mediterranean-type climate and socioeconomic characteristics, Portugal faces increased health risks, with projected temperature rises of 2.5 °C to 4 °C by the end of the century [3].

In light of these challenges, health professionals have an essential role in formulating strategies adapted to specific socio-ecological environments to reduce the impact of and vulnerability to CC and increase the resilience of populations [2]. They are well-informed, trustworthy, and in a solid position to communicate the risks CC poses and the benefits of adaptation [4,5]. In this context, studying physicians' perceptions of CC in the ecological transition is justified by the relevance of these professionals in public health [6]. Therefore, research documenting their attitudes and knowledge can provide valuable insights to drive concrete and informed actions toward a healthier and more sustainable future for all.

There are few studies focusing on physicians' perceptions of CC, and none focused on Portuguese physicians [[7], [8], [9], [10], [11], [12], [13], [14], [15], [16], [17], [18], [19], [20], [21]]. Most studies of physician perceptions utilized quantitative methodology, with the exception of Boer [9], who used a qualitative approach. The current study bridges the contextualized health professional perspective gap by exploring how Portuguese health professionals perceive climate change and mitigation, particularly in the defined context of their practices. It also enables insights into potential recommendations for health policy strategies, specifically regarding awareness-raising and education plans for these professionals.

Thus, this analysis focuses on the perceptions of CC in a small group of physicians whose expressed positions are influenced by both lay and professional worlds [2,4,6,7,22]. Our object of study is the perceptions of physicians practicing medicine in Portugal about the impact of CC on health. Hence, the general objective is to know and understand how physicians understand, explain, and experience the impacts of CC on health in their clinical practice and, as a specific objective, to ascertain their level of knowledge on this subject and what kinds of educational opportunities might be useful.

## 2 Methods

### 2.1 Study design

In line with our focus on social sciences, interviews utilized an objective script to attempt to understand the opinions of the participant group, with the interviewer having no prior personal or professional affiliations that could have influenced the results. A qualitative research approach was used to gain insights into individuals' firsthand experiences, focusing on their thoughts, actions, values, representations, beliefs, opinions, attitudes, and habits [23]. The case study strategy was employed to establish boundaries, delimiting the phenomenon within its context for a detailed and in-depth analysis [23]. We choose a constructive approach as the best method for fully grasping participants' perspectives, experiences, and interpretations without imposing biases. This is facilitated through open dialog and interaction [21]. Because perceptions of physicians regarding the health implications of CC has not been studied extensively, this research took an exploratory approach to become more familiar with the issue and make it more explicit [24].

### 2.2 Data collection

Data were gathered using semi-structured interviews with open-ended questions (Table 1). Based on prior studies, we formulated an interview script to address the research objectives [9,11,15,19]. The script was reviewed by colleagues knowledgeable in qualitative research methods to verify the validity in the context of Portuguese physicians. We tested the script, ensuring the participants’ clarity and adequate comprehension. Participants had the freedom to interpret the scope of the questions, which facilitated a more comprehensive reflection of their perceptions and experiences. After the interviews, we reviewed the transcripts with participants to ensure accuracy and to allow participants to verify the researcher's understanding of their responses and add any necessary clarifications. Additionally, we applied a closed-ended survey (Supplementary Material A) to perform a sociodemographic characterization of the participants (Table 2). The corresponding author conducted each interview to ensure consistency and rigor. We held the interviews individually (one-on-one) to create a comfortable environment for participants to respond to the questions. At the beginning of each interview, we reiterated the research's purpose. We acquired oral and written consent from participants for a recorded interview and registered the audio using a tablet. The interviews took place between October 12 and November 2, 2022, with participants choosing the format: face-to-face (*n* = 2), telephone (*n* = 7) or videoconference (*n* = 4). The meetings lasted an average of 30 min, the shortest being 17 min (face-to-face) and the longest 43 min (Zoom).

**Table 1:** Semi-structured interview guide.

Objectives	Dimensions	Questions	Guiding questions
1: To know and understand perceptions of climate change (CC)	Perceptions of CC	What is CC?	
		What are the causes of CC?	Do you believe that humanity has contribute to CC? How?
			Do you know some of the impacts of CC?
		Personally, are you concerned about CC? Why?	
		What behaviors do you adopt in your daily life to prevent and minimize the impacts of CC?	
		Do you think the organization where you work cares about CC? How?	
2: To know and understand the perceptions of CC on health	Health impacts of CC	Do you believe that climate change has any health consequences?	If the answer is no, ask why
			Ask what these health consequences are and to identify and explain them.
			Ask if they have witnessed these impacts in their professional practice.
		Personally, are you concerned about the impact of CC on health? Why?	
3: To know and understand the knowledge they have about impacts of CC on health and if they feel the need to obtain training in this subject.	CC information and training	How well informed do you think you are about the association between climate change and health impacts?	
		Where and when did you obtain this information / knowledge?	
		Do you think that physicians should get specific training on CC and its effects on health for clinical practice, i.e. know what the impacts of CC are on health: either in terms of morbidity or mortality? Why?	
		Do you believe that this training should be included in the basic curriculum of medical courses?	Why?

**Table 2:** Demographic data of the participants in the study.

Code	Sex	Age	Medical specialty	Years in the profession	Type of workplace		District
E1	F	29	General and Family Medicine - Internal	4	Healthcare Center	Public	Porto
E2	F	37	Reconstructive Plastic Surgery	12	Hospital	Public	Porto
E3	F	46	General and Family Medicine	22	Healthcare Center	Public	Porto
E4	F	29	General and Family Medicine - Internal	3	Healthcare Center	Public	Porto
E5	F	73	Occupational Medicine	43	Private Company	Private	Aveiro
E6	M	69	Internal Medicine	44	Hospital	Private	Porto
E7	M	48	Pulmonology	23	Hospital	Private	Porto
E8	M	55	Pulmonology	30	Hospital	Public	Coimbra
E9	F	33	General and Family Medicine- Internal	3	Healthcare Center	Public	Porto
E10	F	28	General and Family Medicine - Internal	2	Healthcare Center	Public	Aveiro
E11	M	39	General and Family Medicine	7	Healthcare Center	Public	Porto
E12	F	35	Internal Medicine	10	Hospital	Public	Porto
E13	M	45	Internal Medicine	21	Hospital	Private	Porto

**Note:***M* = masculine; *F* = feminine. We had 8 women (68 %) and 5 men (38 %). This aligns with national statistics, which show that there are more women than men in the medical profession (according to the Portuguese Medical Association there are 57 % women and 43 % men in the medical profession) [40].

Answers were analyzed by including a description and interpretation of individual constructions and a comparison between the interviewees and those of the researchers and available applicable theories [23,24].

### 2.3 Ethics

This study followed ethical procedures outlined in national and European codes of ethics, ensuring adherence to principles of informed consent and anonymization. The Open University Scientific Council approved this qualitative research.

### 2.4 Sample and recruitment strategy

Local health units in Porto, Portugal were contacted via email in the last week of September 2022 and these provided email contacts of potential participants. Selection criteria were availability, willingness to participate, and practicing medicine in Portugal. Candidates were sent all relevant information to facilitate the decision to participate in this research. Since physicians constitute a challenging demographic group, at the end of participant interviews we also requested recommendations of other colleagues to join the study, so snowball sampling was applied [25]. This resulted in participants from three different districts. In total, we contacted 20 physicians, and 16 responded. Two were scheduled for interviews but did not show up, and one interview was eliminated because the recording was unsuitable for transcription. The physicians who refused to participate came from same specialties and had similar demographic characteristics to those who were interviewed. We applied theoretical saturation, obtaining as many interviews as possible until we reached the point where the data collected no longer provided additional insights into the research question, and this was reached with our sampling of 13 participants [26].

### 2.5 Data analysis

The technique chosen to interpret the interviews in this study was content analysis [27]. The content analysis consisted of categorizing and ordering the content based on our research objectives, theoretical framework, and interview data, creating a categories grid (Supplementary Material B). In this process, we transcribed the interviews using Microsoft Word 365, reviewed the transcripts for accuracy, and then made a vertical analysis of each interview and a horizontal analysis of all interviews, considering the indicators and issues established by our research objectives, theoretical framework, and interview data. Words aligned with the study's objectives were highlighted ("registration units") and used as the essential content for analysis. Then, in a final reading, excerpts were highlighted, checking that they could be grouped into other categories, emerging subcategories that allow for more precise and accurate categorization. The most extended content, such as sentences, was considered to ensure the analysis's fidelity and validity. We use MAXQDA 2020, a qualitative analysis program, for all these procedures.

## 3 Results

Participants included 8 women and 5 men, with ages ranging from 28 to 73, and professional experience ranging from 2 to 44 years. This group came from five medical specialties: Reconstructive Plastic Surgery, Occupational Medicine, General and Family Medicine, Internal Medicine, and Pulmonology. Their workplaces were diverse, with nine working in the public sector, namely six in Porto and Aveiro local health units and three in Porto and Coimbra hospitals. The remaining four participants work in the private sector, namely three in hospitals in Porto and one in a company in Aveiro. Table 2 shows the complete demographic data of the study participants.

Table 1 shows the objectives and the open questions asked of the participants. From the answers, three main categories emerged during the content analysis, with results described below in the following sections: (3.1) perceptions of climate change, (3.2) health impacts of climate change, and (3.3) climate change information and training.

### 3.1 Perceptions of climate change

The participants in this study exhibited diversity in their understanding of climate change. Of the 13 participants, 4 described CC by its consequences, 4 connected it to climate variation, and 4 linked it to global warming. Only 1 participant described CC by its causes, stating that it is related to human activity.

Concerning the causes of CC, all participants stated that human activity is the primary contributor. They mentioned that the main factors responsible were greenhouse gas emissions, fossil fuels, and excessive production. Also 8 of the participants mentioned natural causes along with anthropogenic causes, recognizing that CC may affect climate patterns, but isolated and extreme events may not necessarily be of human origin but rather part of natural phenomena, as can be seen in the example excerpt.

*These are changes caused by human events or related to human activity. However, we know that some climate changes are obviously also of earthly origin, such as thunderstorms, volcanic activity, and some forest fires that man does not cause [E8].*

As for the consequences of CC, Table 3 shows that heat and cold waves were the most cited, followed by extreme weather events, floods and droughts. Regarding concern about the consequences of CC, 9 participants expressed worry about these impacts, particularly those harming their descendants. As detailed in the following excerpt, the concern is future-oriented, not immediate.

I worry, but at the same time, I don't worry. I worry about my nephews—I don't have children, but nephews—and they will have to live in a more difficult world, but at all levels [E3].

**Table 3:** Identifying the impacts of climate change.

The impacts	No. of occurrences
Heat waves	10
Waves of cold	7
Increase in extreme events	7
Floods	6
Droughts	5
Defrosting	5
Wildfires	4
Airborne dust	4
Storms	4
Decrease in biodiversity	3
Hurricanes	2
Tornado	2
Change in food production	2
Heavy rains	2
Water shortage	2
Cyclones	1
Change of seasons	1
Changes in temperatures	1
Migrations	1
Poverty	1

**Note:** Each respondent recognized multiple items (multiple responses).

With slightly different opinions, 3 interviewees expressed concern regarding the immediate impacts of what we can do now to minimize the effects of CC, as the following statement illustrates.

I worry about climate change, of course. But I am much more concerned about finding out in my day-to-day life what I can do to avoid waste and to save energy in the broad sense [E7].

Also noteworthy was the reaction of 1 participant, who expressed a more holistic concern, warning of the dangers that some backward-looking policies, namely the burning of fossil fuels, could pose in the near future.

So, when we start to see the prominent environmental summits, (…), they're stuck in the middle, aren't they? Moreover, this extreme event, the war in Ukraine, is also going to happen because of the imminent energy risks associated with the interruption of gas supplies from Russia. Unfortunately, it will give significant support to those who still like to burn fossil fuels. So here it is, (…), back to forms of energy that should have been completely banned. So, there is now a political excuse to go back many years. So, my outlook for the next few years is not good [E8].

In Table 4, it is possible to identify the behaviors that participants say they have adopted to minimize the impacts of CC. Recycling, moderating consumption, and saving water and energy were the most frequently mentioned. Finally, we asked them if they thought the organization they worked for was concerned about CC. Eight participants said the organizations behave positively in this context, with recycling, energy saving, and environmental certification being the most cited. Two of the participants mentioned measures taken to minimize the impact of CC on health:

*So, there is some concern, particularly about the well-being of workers in some circumstances. (…).*Some companies are significantly concerned about starting to equip their industrial ships with cooling systems; as you know, the vast majority of machinery in the industry has higher temperatures [E8].

*And have the logistics to deal with and respond to new and urgent illnesses that may be a consequence of climate change [E6].*

**Table 4:** Behaviors participants have adopted to minimize the impacts of climate change.

Behaviors adopted	No. of occurrences
Recycling	11
Moderation in consumption	7
Water saving	5
Energy saving	5
Use of biological products	2
Shopping locally	2
Change of energy	2
Use of public transport	2
Reduction in meat consumption	1
Reduction in the use of plastics	1
Walking in nature	1
Short baths	1

**Note:** Each respondent recognized multiple items (multiple responses).

On the other hand, 5 participants rated their workplaces negatively regarding attitudes toward combating CC, mentioning an excessive waste of paper and other resources and high energy consumption.

### 3.2 Health impacts of climate change

All 13 participants acknowledged climate change's impact on human health, highlighting respiratory, infectious (vector-borne), and psychological diseases. Table 5 details additional health concerns raised, and 3 participants observed a rising mortality trend due to climate change impacts and anticipated its persistence. Another pointed out increased hunger (malnutrition) from CC-induced droughts and reduced food production.

**Table 5:** Identification of diseases associated with climate change.

Diseases	No. of occurrences
Respiratory	11
Infectious	6
Vector-borne	5
Psychological	5
Cardiovascular	5
Oncological	4
Mortality	3
Skin aging	2
Dehydration	2
Hunger/malnutrition	1
Sleep disorders	1
Digestive	1
Epidemics	1
Decreased fertility	1

**Note:** Each respondent recognized multiple items (multiple responses).

Mortality it is not a disease, but it was identified as a health consequence.

Although all the interviewees were aware of the impact of CC on health and knew how to identify some specific diseases, some said that in their professional practice, they need help to make this connection, i.e., to establish whether this or that disease resulted from CC.

*It's very difficult to define, isn't it? But sometimes, we deal more with occupational illnesses from exposure or miners’ exposure, which does not really have to do with climate change [E1].*

Concerning witnessing the impacts of CC at work, 8 participants confirmed that they had witnessed the worsening of some illnesses, particularly respiratory illnesses, due to excessive heat or cold and fires. One participant highlighted cases of hypothermia due to the excessive cold in winter and inadequate housing conditions.

*In a hospital emergency room, I have had several cases of elderly people with significant hypothermia in the winter months - I am talking about Coimbra (center), not Bragança (north). (…) Moreover, it was not an accident; it was not because they had fallen into the sea or a swimming pool, nothing like that; it was exposure to cold in their homes [E8].*

However, 2 participants mentioned that they had never witnessed these impacts during their work because they had only been working for a few years, and another professional with many more years of experience also indicated that she had never observed the effects of CC on the health of her patients.

Regarding concerns about the impact that CC can have on human health, 8 interviewees were apprehensive about effects on their patients' health and the worsening of certain diseases. Three interviewees were more dissatisfied with the consequences "for society," warning that this issue will have repercussions in the future, while 2 participants said they were not very concerned about the health impacts but rather those affecting the planet, as the following speech illustrates.

*I sometimes forget more about this impact on health because the truth is that climate change is at a level where it is a concern. There will not even be a future because of climate change, right? [E1]*

### 3.3 Climate change information and training

Five interviewees considered themselves "poorly informed,” 5 described themselves as "relatively informed”, and 3 participants believed they are well-informed on CC. Two of these were pulmonologists, which may influence their knowledge in this area because they are the ones who typically deal with respiratory diseases.

Participants reported that their sources of information about the impacts of CC on health included the media, such as television, websites (WHO, TED Talks, and the European Respiratory Society), and social networks. One also mentioned the website of the Directorate General for Health, 2 had read scientific articles on the subject, and 1 stated that she acquired her knowledge through professional practice.

The majority of the participants believed that it is essential to have training in the medical course on the impacts of climate change on health. In contrast, 2 interviewees mentioned that individuals who understand the subject should handle the issue of climate change rather than physicians. The 11 participants who concurred that training on climate change's impact on health should be part of the medical course provided the rationale that to make a good diagnosis, one needs to know people well, "take into account the circumstances in which they live and what ails them," [E6] and "understand what dimension the person lives in" [E4]. It is "important to be aware of what can influence or cause the pathology" [E10].

With an opposing view, 2 participants felt it was inappropriate to have this training during medical education, as we see in the following excerpt.

It is inappropriate in basic training (medical school). If we want to know everything in basic training, we must have a 20-year course. (…) Later on, yes, yes. Yes! I think so, but it is optional [E11].

## 4 Discussion

The objective of this qualitative study was not to generalize the results to a larger population but rather to identify and explore the diverse perspectives and experiences of the participants. Therefore, it is important to note that the findings are context-specific and may not be directly applicable to other settings or populations. Instead, our focus is on generating detailed descriptions that contribute to a deeper understanding of the phenomenon being studied.

As far as we know, this is the first attempt to describe how physicians practicing in Portugal understand, explain, and experience the impacts of CC on health. The insights gained from the 13 interviews with physicians working in the Aveiro, Coimbra, and Porto districts offer valuable cues for shaping future research in this critical domain.

Participants clearly understood CC effects and causes, aligning with previous studies involving health professionals [10,11,15]. All participants expressed apprehension about the consequences of CC, mirroring concerns shared by the wider Portuguese population [28]. For the majority, the paramount worry revolves around the future and what kind of planet we will leave for future generations. This temporal perspective aligns with the notion that CC consequences may not be immediately perceptible, extending beyond the layperson's immediate awareness, as articulated by Beck and supported by other studies [[29], [30], [31], [32]]. This lack of awareness of the consequences of CC in the present may have implications for identifying symptoms in physicians’ daily practice. On the other hand, concern for future generations is one of the reasons that most motivate people to act in the face of CC [33].

As for personal actions and those of organizations to combat CC, recycling and moderating consumption were the most cited, which have a relatively small impact on mitigating CC [34]. The attitudes toward changing energy and using public transport, which are more important in contributing to reducing GHGs, were the least mentioned in this research. These measures require the most significant lifestyle changes, which may explain why several studies demonstrated similar results [28,34].

All participants agreed that CC impacts human health and they can identify various illnesses that may worsen or appear because of the phenomenon. They also indicated that CC affects people's mental health and well-being, dimensions which, despite an increase in scientific production on the implications of CC for mental and psychosocial health, are still not adequately understood and addressed [14].

In addition, more than half of the interviewees have already witnessed these effects in their clinical practice. In particular, they have seen increased respiratory problems caused by pollution, excess heat and cold, and fires. This is consistent with other studies showing that physicians report that their patients already suffer from the health consequences of CC, including the appearance and exacerbation of respiratory diseases due to increased pollution, thermal extremes, forest fires, pollen spread, fungi, and infectious agents, emphasizing the severity and importance of this issue [15,17,21,35,36].

Every participant conveyed a shared concern about CC. However, a few exhibited limited concern about its impact on health or prioritized other consequences. This observation suggests that some individuals may not have directly encountered the effects of climate change in their clinical practice, resulting in a disconnect between health issues and climate change. Additionally, the need for more training and information among participants about the effects of climate change on health may contribute to this disconnect [37,38].

A significant number of participants expressed a need for a better understanding of the correlation between CC and its' impact on health. They emphasized the importance of incorporating training on this topic into medical education courses. Additionally, participants highlight the necessity for increased knowledge to effectively communicate with patients about the advantages of modifying behaviors for personal health and the environment. This knowledge gap hampers their ability to establish a clear connection between climate change and their patients' health. Similar sentiments are echoed in other studies, emphasizing the widespread need among physicians for more information on the links between climate change and health [9,11,12,15,17,21,39]. These studies also stress the importance of enhanced knowledge for improving patient communication regarding climate change and its health implications.

Our participants identified television and the internet as their primary sources of information on CC. The preference for the internet aligns with existing trends, as noted by researchers Lewandowski et al., who emphasize the significance of blogs for scientific discussions on CC [37]. An interesting observation emerged regarding physicians' reliance on television, underscoring the potential impact of television in shaping healthcare professionals' comprehension and communication strategies concerning climate-related health issues.

Most participants suggested that medical training should incorporate this knowledge to better inform patient communication and promote behavior changes for improved health and environmental outcomes, emphasizing the importance of education and awareness among physicians and the broader population while navigating the potential pitfalls of disinformation in the digital age. Incorporating these findings into public health programs may be extremely effective in tackling the effects of climate change on health, by tailoring communication strategies to specific target populations, focusing on preventative actions and health-promoting behaviors. Another pivotal aspect is to integrate the findings into healthcare professionals' training programs to improve their understanding of the links between climate change and health and provide guidance on how to effectively communicate with patients about climate-related health risks and precautions. All of these projects should be supported by partnerships with local communities to design and implement initiatives that address climate-related health risks. This could involve providing resources to vulnerable groups, organizing community workshops on climate resilience, or developing support networks for people suffering from climate-related health conditions.

This study had some limitations. There is a notable scarcity of scientific literature and standard methods focused on CC perceptions among health professionals, particularly physicians.. Though limited by a small number of participants, the approach taken in this study allows for a thorough exploration of participants' perspectives within the defined context. Despite potential limitations in generalizability, the research provides insights about participants' perceptions of CC, and may inform recommendations for health policy strategies, particularly in awareness-raising and education plans for healthcare professionals.

## 5 Conclusion

This study is the first to delve into how physicians in Portugal perceive and experience the impacts of CC on health, including mental health, a dimension often overlooked in existing literature. Despite being aware of CC-related health effects, the majority of the participants revealed difficulties in identifying CC impacts on health in their daily practice, which indicates a need for increased awareness among healthcare professionals regarding the connection between CC and health to avoid repercussions on diagnosis and preparedness for the impacts of extreme climate events.

Studying how well physicians and other health professionals understand the health impacts of climate change, when their insights can significantly aid in creating urgent strategies to shift individual behaviors, will be important for achieving an ecological transition and lessening the impacts of global warming. Simultaneously, healthcare providers can shape the narrative around climate change mitigation and adaptation actions as opportunities that benefit individual and public health.

## Funding

This work was supported by the R&D Unit Centre for Functional Ecology—Science for People & the Planet (CFE), with reference UIDB/04004/2020 and DOI identifier https://doi.org/10.54499/UIDB/04004/2020 financed by FCT/MCTES through National Funds (PIDDAC) with and extension at the 10.13039/100010040University Aberta, and the Associate Laboratory TERRA, with reference LA/P/0092/2020.

## CRediT authorship contribution statement

**Nidia Ponte:** Writing – original draft, Visualization, Validation, Software, Project administration, Methodology, Investigation, Formal analysis, Data curation, Conceptualization. **Fátima Alves:** Writing – review & editing, Visualization, Validation, Supervision, Methodology, Formal analysis, Conceptualization. **Diogo Guedes Vidal:** Writing – review & editing, Visualization.

## Declaration of competing interest

The authors declare that they have no known competing financial interests or personal relationships that could have appeared to influence the work reported in this paper.

## Appendix Supplementary materials

Image, application 1

Image, application 2
